# Effects of remote ischemic preconditioning on coronary blood flow and microcirculation

**DOI:** 10.1186/s12872-023-03419-0

**Published:** 2023-08-17

**Authors:** Zhen-Zhou Zhao, En Li, Xue-Jie Li, Quan Guo, Qing-Bo Shi, Mu-Wei Li

**Affiliations:** Heart Center of Henan Provincial People’s Hospital, Fuwai Central China Cardiovascular Hospital, Henan Province, Zhengzhou, 450003 Henan Province China

**Keywords:** Coronary circulation, Quantitative flow ratio, Microcirculatory resistance, Coronary angiography

## Abstract

This study aimed to determine the effect of short-term remote ischemic preconditioning (RIPC) on coronary blood flow and microcirculation function using the quantitative flow ratio (QFR) and index of microcirculatory resistance (IMR). We randomly divided 129 patients undergoing coronary angiography (CAG) into RIPC and control groups. Following the first CAG, we randomly divided the patients further into the unilateral upper limb and lower limb groups for four cycles of ischemia/reperfusion circulation; subsequently, we performed the second CAG. During each CAG, contrast-flow QFR (cQFR), fixed-flow QFR (fQFR), and IMR (in patients with cardiac syndrome X) were calculated and compared. We measured 253 coronary arteries in 129 patients. Compared to the control group, the average cQFR of the RIPC group increased significantly after RIPC. Additionally, 23 patients with cardiac syndrome X (IMR > 30) were included in this study. Compared to the control group, IMR and the difference between cQFR and fQFR (cQFR-fQFR) both decreased significantly after receiving RIPC. The application of RIPC can increase coronary blood flow and improve coronary microcirculation function.

## Introduction

Ischemic preconditioning is an adaptive response of tissues to transient ischemia, which can lengthen the duration of tissues’ tolerance to acute ischemia [1]. Repeated short-term coronary artery occlusion/reperfusion could significantly reduce the area of subsequent myocardial infarction in dogs [2]. However, regional ischemic preconditioning of the coronary artery in patients with coronary artery disease (CAD) is difficult to achieve. Remote ischemic preconditioning (RIPC) has been reported to protect distant organs without causing a direct injury to the target organs (e.g., the heart or brain). However, the research results on RIPC have been controversial. RIPC was reported to reduce the infarct size and improve the prognosis of patients with myocardial infarction [3–6]; although, a few studies have achieved neutral results [7]. Studies have reported that RIPC could decrease the ischemia-reperfusion injury (IRI) after vascular recanalization, and the function of coronary microcirculation plays an important role in the causation of IRI. Therefore, we speculated that RIPC can increase coronary blood flow and improve coronary microcirculation function. Currently, the degree of ischemia in epicardial vessels is usually assessed by calculating the flow reserve fraction (FFR). Based on the FFR, the quantitative flow ratio (QFR) is an advanced analysis system without a guidewire, which can accurately and efficiently analyze the function of stenotic coronary arteries, which can be obtained at the same time as a diagnostic coronary angiography (CAG) is performed on the patient. QFR can be obtained from two different flow models: fixed-flow QFR (fQFR) and contrast-flow QFR (cQFR) [3, 4], and cQFR-fQFR can be confirmed to evaluate the coronary microcirculation function [5]. The index of microcirculatory resistance (IMR) is the product of remote coronary artery pressure and the average conduction time in a state of maximum hyperemia. It is an objective quantitative index used to evaluate and calculate coronary artery microcirculation, and IMR > 25U indicates impaired coronary microcirculation [6]. Our study aimed to accurately analyze the alterations of coronary artery blood flow in patients with CAD and microcirculation function in patients with cardiac syndrome X (CSX) after short-term RIPC by cQFR, cQFR-fQFR, and IMR.

## Methods

From September 2021 to February 2022, 129 patients in our institution were enrolled and randomly divided into the RIPC and control groups. In addition, each group was randomly divided further into the upper limb (UL) and lower limb (LL) subgroups. The inclusion criteria were as follows: (1) age ≤ 80 years and age ≥ 18 years; (2) requiring CAG; (3) left ventricular ejection fraction > 50%; (4) normal preoperative creatinine kinase-MB and cardiac troponin T level. The exclusion criteria were as follows: (1) age > 80 years or age < 18 years; (2) history of coronary artery bypass grafting; (3) acute coronary syndrome within 1 month; (4) long-term poorly controlled hypertension (systolic blood pressure > 180 mmHg or diastolic blood pressure > 110 mmHg or systolic blood pressure < 90mmHg or systolic blood pressure < 60mmHg) or poorly controlled heart rate (heart rate > 100 bpm or heart rate < 50 bpm); (5) cardiac valve diseases, congenital heart diseases, or severe arrhythmias; (6) deep venous thrombosis or thrombophlebitis of extremities; (7) history or existing severe trauma of the extremities; (8) active bleeding, peptic ulcer, blood coagulation dysfunction, cerebral hemorrhage, or craniotomy within 6 months; (9) poor systemic conditions, infection, severe hepatic and renal insufficiency, malignant tumor, or cachexia; (10) Creatinine clearance < 30 mL/min (or eGFR < 30 mL/min/1.73 m2), or renal insufficiency requiring dialysis; 11) Active liver disease or elevated persistent ALT or AST ≥ 3 x ULN; 12) history of alcoholism, substance abuse; and unable/unwilling to abstain from alcohol and stop substance abuse during the study; 13) have received a major organ transplant (e.g., lung, liver, heart, bone marrow, kidney); and 14) left coronary artery trunk or right coronary artery orifice lesions, excessive or severe tortuous overlap of target vessels, diffuse lesions of remote target vessels, or coronary artery with low quality CAG images (to ensure the accuracy of the QFR measurement). The definition of the diseased vessels was adopted from the current international diagnostic criteria for coronary heart disease (vascular stenosis > 50%), and the degree of stenosis was independently evaluated by two experienced interventional physicians. CSX was diagnosed according to the diagnostic criteria for microvascular angina in 2018 [8]: symptoms of myocardial ischemia, absence of obstructive CAD (< 50% diameter reduction by CAG), objective evidence of myocardial ischemia, and evidence of impaired coronary microvascular function (IMR > 30).

The RIPC group included 71 patients, wherein 68 vessels were analyzed (eight right coronary arteries [RCAs], 32 left anterior descending arteries [LAD], and 28 left circumflex arteries [LCXs]) of 39 patients in the UL group and 63 vessels (eight RCAs, 28 LADs, and 27 LCXs) of 32 patients in the LL group. We randomly selected 58 patients for the control group, including 64 vessels (seven RCAs, 29 LADs, and 28 LCXs) of 30 patients in the UL group and 58 vessels (10 RCAs, 25 LADs, and 23 LCXs) of 28 patients in the LL group.

This study was approved by the institutional ethical board [Approval Number:(2021)(13)], and written informed consent was also obtained from each participant,and this study is registered in ClinicalTrials.gov (Tag: NCT04766749).

The RIPC instrument used in this study was the Yijiabao ischemic preconditioning therapeutic instrument, independently developed by the China Hongjing Medical Company. According to the standard CAG procedure, all patients received standardized treatment before the procedure, and two experienced clinicians performed CAG; IMR was further measured in patients without obvious vascular stenosis. Subsequently, those in the experimental group received four cycles of RIPC in the left upper or lower limb after completing the first CAG. The RIPC procedure comprised 5 min of compression and ischemia followed by 5 min of relaxation and reperfusion, and the cuff of the RIPC instrument was pressurized to 200 mmHg. The same therapeutic device was placed on the left upper or lower limb of patients in the control group, and the cuff was tied but not inflated. Immediately after completing RIPC, the second CAG (and IMR) was performed in the same position as the first.

The AngioPlus system (Pulse Medical Imaging Technology, Shanghai, China) was used to calculate the QFR. We chose two planar angiographies close to the standard position and eliminated collateral interference, and the separation between the two planes was at least 25°. The three-dimensional reconstruction of the angiographic results was performed using the AngioPlus system. The software computed the following two QFR pullbacks: (a) fQFR pullback, a fixed empiric hyperemic flow velocity of 0.35 m/s derived from previous FFR studies was used for computation; and (b) cQFR pullback, a frame count analysis performed without pharmacologically-induced hyperemia and the modeled hyperemic flow velocity, which was derived according to the frame count used for computation [8]. The calculation of QFR was performed independently by two experienced operators who were blinded to the clinical grouping.

At the end of CAG, IMR was measured in patients with CSX with a thermodilution technique as described previously [9]. Briefly, the aortic pressure transducer was zeroed to air, and after routine preparation and calibration, the pressure wire was introduced in the guide catheter and positioned with the wire sensor at the guide tip for electronic equalization. The pressure wire was advanced to the distal part of the coronary artery (approximately two-thirds of the vessel). After intracoronary injection of 250 mg of isosorbide dinitrate, the following parameters were measured, both at baseline and after hyperemia was induced with intravenous infusion of adenosine at a rate of 140 mg/kg/min: (1) mean aortic pressure, (2) mean distal pressure, and (3) mean transit time. The mean transit time was calculated as the average of three transit time measurements during three separate injections of 3 ml 0.9% saline at room temperature. IMR was then calculated as the mean distal pressure at hyperemia multiplied by the mean transit time at hyperemia. Equally, the calculation of IMR was also performed independently by two experienced operators who were blinded to the clinical grouping.

SPSS25.0 was used to analyze the data. The data of continuous variables were expressed as means ± standard deviations or medians and interquartile ranges. The statistical description of classified variable data was expressed as quantities and percentages, and the differences between the groups were compared using the chi-square test. For data conforming to normal distribution, one-way anova was used for comparison between groups. The Kruskal-Wallis test was used to compare data that did not conform to the normal distribution. Two-tailed tests were used for statistical analysis, with *P* < 0.05 being statistically significant.

## Results

As shown in Table 1, a comparison of baseline characteristics among the RIPC and control groups demonstrated no statistically significant differences in age, sex, body mass index, hypertension, hyperlipidemia, diabetes, smoking, drinking, medication, or the left ventricular ejection fraction. No significant differences in the coronary anatomical distribution or coronary lesion features among the four groups were observed (Table 2).

**Table 1:** Clinical features of the 4 groups

	RIPC UL (n = 39)	RIPC LL (n = 32)	control UL (n = 30)	control LL (n = 28)	test statistic	*P* value
Male[n(%)]	33(84.6)	27(84.4)	18(60)	23(82.1)	7.765	0.051
Hypertension[n(%)]	25(64.1)	18(56.3)	16(53.3)	21(75.0)	3.488	0.322
Diabetes[n(%)]	3(7.7)	7(21.9)	4(13.3)	5(17.9)	3.103	0.376
Smoking[n(%)]	18(46.2)	16(50)	12(40)	11(39.3)	0.986	0.805
Dyslipidemia[n(%)]	27(69.2)	23(71.9)	16(53.3)	20(71.4)	3.192	0.363
Age(year)	63(51,65)	61(53,67.75)	63.5(52.25,68)	60.5(55,68.75)	0.047	0.217
BMI	26.6(24.7,27.3)	25.6(24.5,27)	25.3(24.7,26.2)	25.2(24.3,2689)	4.450	0.209
LVEF(%)	59(57,60)	57(55.3,60)	59(55,62)	58(48.5,67)	3.131	0.372

Values are median (interquartile range [IQR] for continuousvariables, and n (%) for categorical variables. BMI: body mass index; LVEF: left ventricular ejection fraction;

**Table 2:** Coronary angiographic characteristics of the 4 groups

		RIPC UL (n = 68)	RIPC LL (n = 63)	control UL (n = 64)	control LL (n = 58)	test statistic	*P* value
target vessel	LAD	32(47.1)	28(44.4)	29(45.3)	25(43.1)	1.352	0.969
	LCX	28(41.2)	27(42.9)	28(43.8)	23(39.7)		
	RCA	8(11.8)	8(12.7)	7(10.9)	10(17.2)		
RVD (mm)		3.25(3.0,3.5)	3.5(3.1,3.5)	3.25(3.12,3.5)	3.25(3.0,3.5)	2.094	0.553
Lesion length(mm)		19.5(14.3,23)	18(13,24)	17(13,21)	20(13,24)	2.346	0.500
DS (%)		76.26(67,87.5)	80(70,86.67)	78.46(68.74,87.29)	74.84(66.15,84.15)	0.780	0.854

LAD: left anterior descending artery; LCX: left circumflex artery; RCA: right coronary artery; RVD: reference vessel diameter; DS: diameter stenosis

**Fig. 1:**
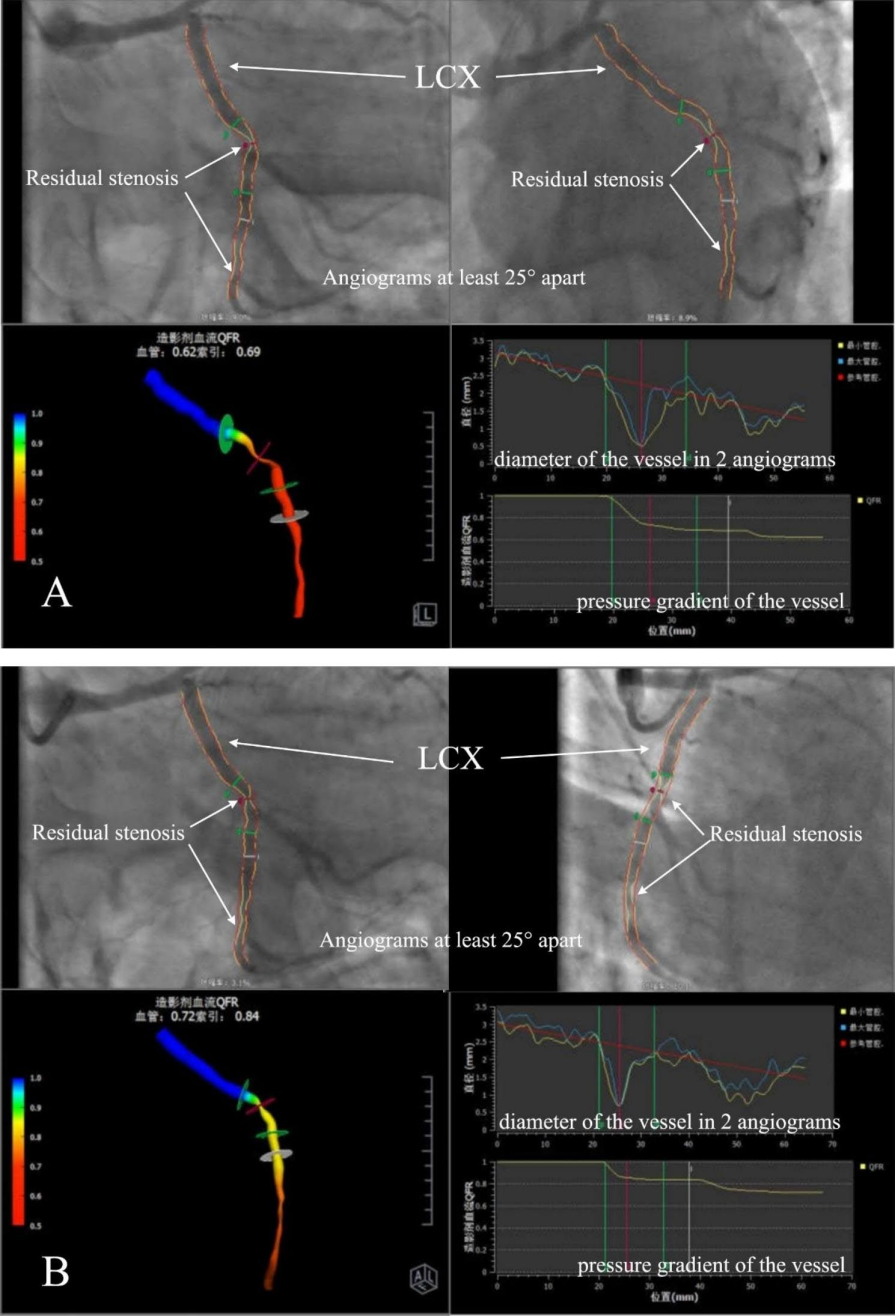
** A diagram of cQFR changes in the experimental group**: A and B represent the cQFR value of the diseased vessel (LCX) before (0.62) and after (0.72) RIPC, respectively. LCX: left circumflex artery

**Fig. 2:**
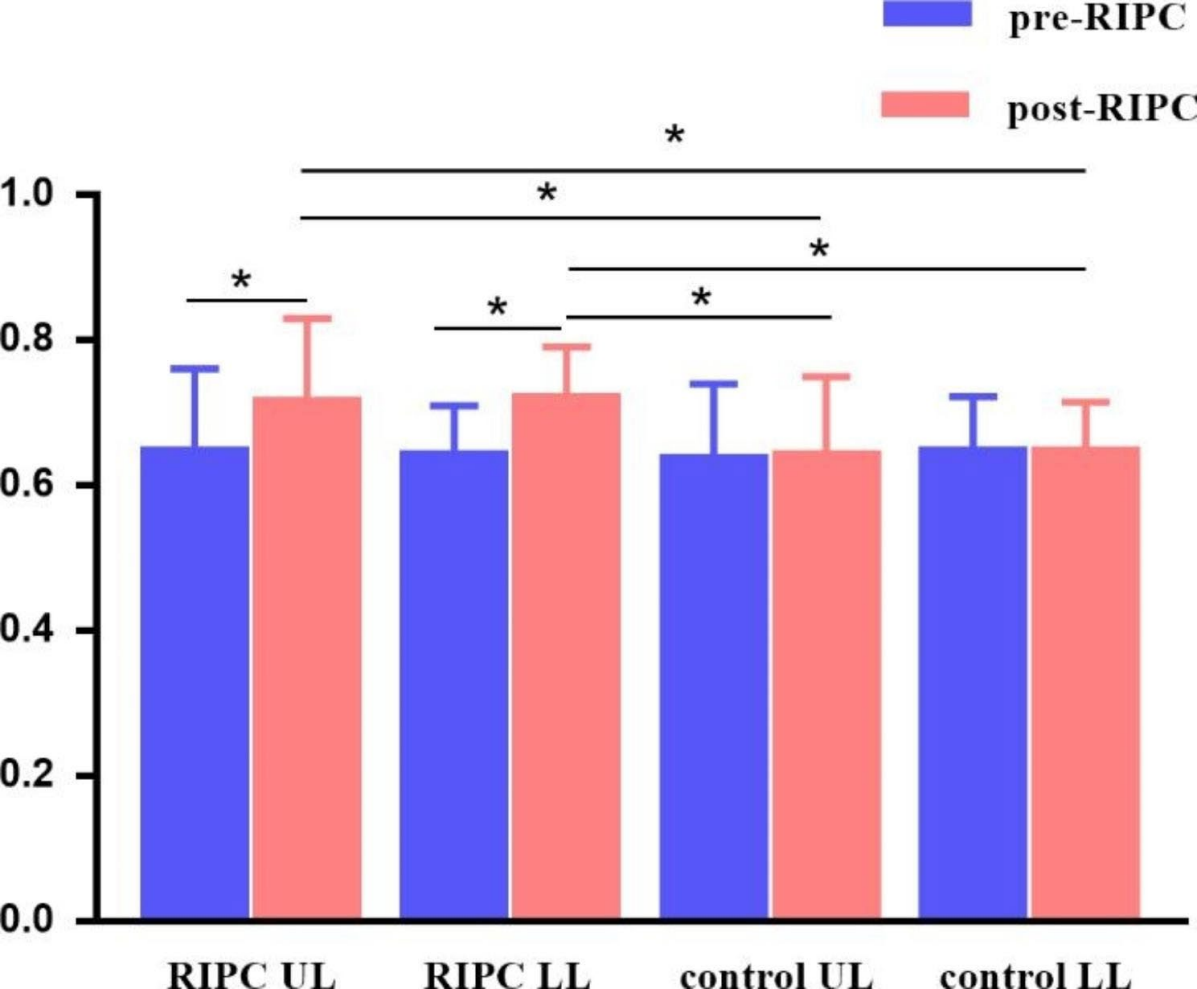
cQFR of pre-RIPC and post-RIPC of the four groups*: Means statistical difference (adjusted *P* < 0.05)

No significant difference in the baseline cQFR between the two groups was observed (*P* = 0.492). The cQFR of the experimental group increased significantly after RIPC (Fig. 1), and no significant difference was observed compared to the control group (*P* > 0.05) (Fig. 2). The cQFR changes in the experimental group were larger than those in the control group (*P* < 0.001). Further analysis revealed that in the RIPC group, the cQFR change in the UL subgroups was lower than that in the LL subgroups (Table 3).

**Table 3:** cQFR findings of the 4 groups

cQFR	RIPC UL (n = 68)	RIPC LL (n = 63)	control UL (n = 64)	control LL (n = 58)	test statistic	*P* value
pre-RIPC	0.645(0.553,0.76)	0.64(0.56,0.71)	0.635(0.573,0.74)	0.645(0.568,0.723)	0.775	0.855
post-RIPC	0.715(0.63,0.83) ^b, c^	0.72(0.65,0.79) ^d, e^	0.64(0.57,0.75) ^b, d^	0.645(0.558,0.715) ^c, e^	125.461	< 0.001
change amplitude	0.07(0.05,0.09) ^a, b, c^	0.08(0.05,0.1) ^a, d, e^	-0.01(-0.02,0.01) ^b, d^	0(-0.02,0.02) ^c, e^	16.485	< 0.001

a: RIPC UL and RIPC LL was significantly different; b: RIPC UL and control UL was significantly different; c: RIPC UL and control LL was significantly different, d: RIPC LL and control UL was significantly different, e: RIPC LL and control LL was significantly different (adjusted *P* < 0.05)

**Fig. 3:**
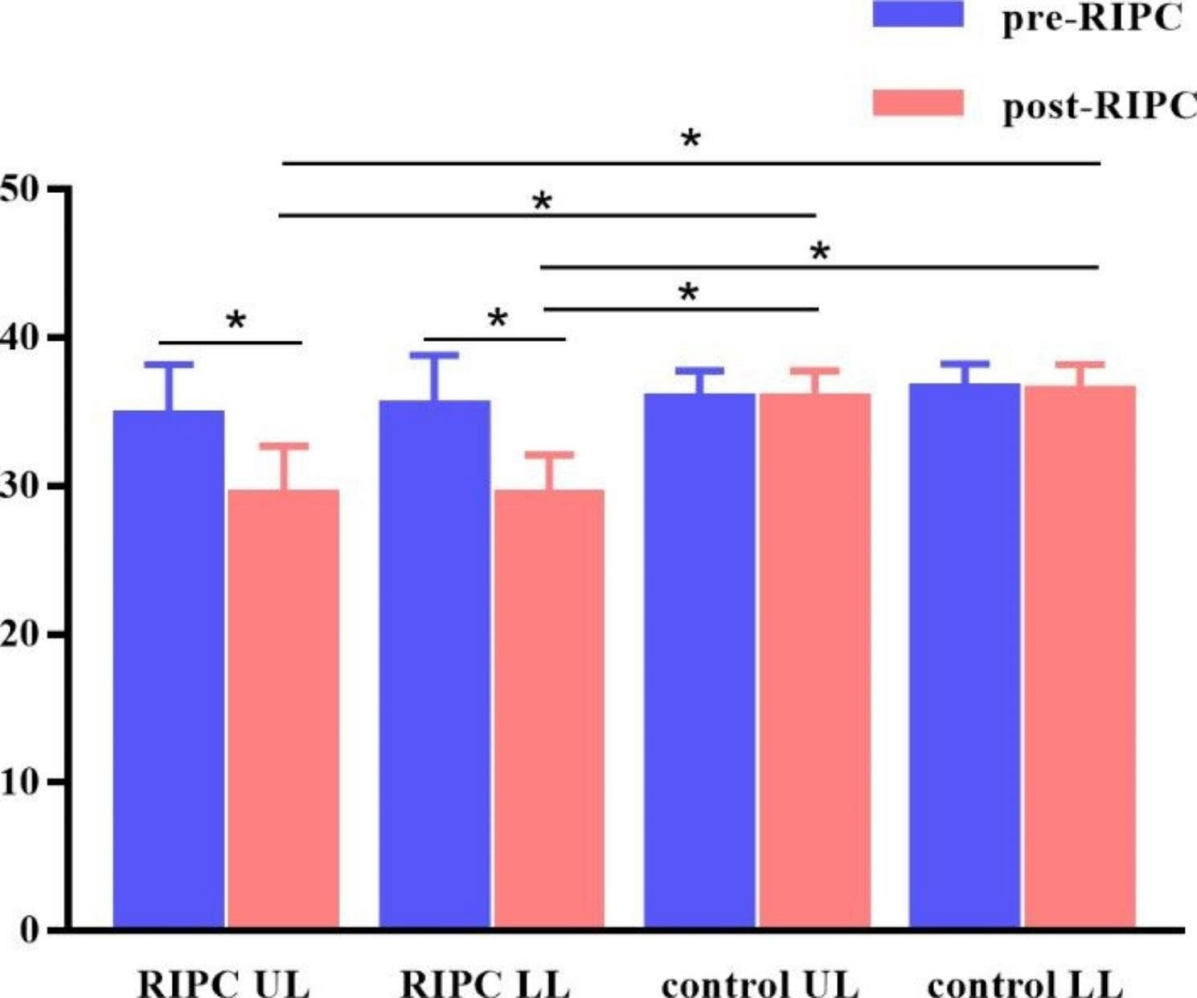
IMR of pre-RIPC and post-RIPC of the four groups*: Means statistical difference (adjusted *P* < 0.05)

A total of 23 patients with CSX were enrolled, including 13 patients in the RIPC group, with 17 diseased vessels (nine vessels of eight patients in the UL subgroup and eight vessels of five patients in the LL subgroup), and 15 diseased vessels of 10 patients in the control group (six vessels of four patients in the UL subgroup and nine vessels of six patients in the LL subgroup). The results demonstrated that IMR decreased significantly in the experimental group after RIPC; no significant difference was observed in the control group (*P* > 0.05) (Fig. 3). Further data analysis demonstrated no significant difference in the decrease of IMR between the UL and LL subgroups of the RIPC group (*P* = 0.555) (Table 4).

**Table 4:** IMR findings of the 4 groups

IMR	RIPC UL (n = 9)	RIPC LL (n = 8)	control UL (n = 6)	control LL (n = 9)	test statistic	*P* value
pre-RIPC	34.67 ± 3.5	35.38 ± 3.42	35.83 ± 1.94	36.56 ± 1.67	0.709	0.555
post-RIPC	29.33 ± 3.35 ^a, b^	29.38 ± 2.72 ^c, d^	35.83 ± 1.94 ^a, c^	36.33 ± 1.87 ^b, d^	18.164	< 0.001
change amplitude	4(3.5,7) ^a, b^	6(4.25,6.75) ^c, d^	0.5(-0.25,1) ^a, c^	1(-0.5,1) ^b, d^	23.967	< 0.001

a: RIPC UL and control UL was significantly different; b: RIPC UL and control LL was significantly different, c: RIPC LL and control UL was significantly different, d: RIPC LL and control LL was significantly different (adjusted *P* <0.05)

No significant differences among the four groups were observed in the basic cQFR-fQFR of the 32 diseased vessels in the above 23 patients with CSX (*P* = 0.925). However, the cQFR-fQFR of the experimental group after RIPC was lower than that of the control group (*P* < 0.001). Further analysis demonstrated no statistical difference in cQFR-fQFR between the UL and LL subgroups of the RIPC group (Table 5).

**Table 5:** cQFR- fQFR findings of the 4 groups

cQFR-fQFR	RIPC UL (n = 9)	RIPC LL (n = 8)	control UL (n = 6)	control LL (n = 9)	test statistic	*P* value
pre-RIPC	0.151 ± 0.028	0.146 ± 0.018	0.145 ± 0.010	0.149 ± 0.014	0.156	0.925
post-RIPC	0.117 ± 0.017 ^a, b^	0.104 ± 0.019 ^c, d^	0.135 ± 0.014 ^a, c^	0.148 ± 0.015 ^b, d^	11.826	< 0.001

a: RIPC UL and control UL was significantly different; b: RIPC UL and control LL were significantly different, c: RIPC LL and control UL was significantly different, d: RIPC LL and control LL was significantly different (adjusted *P* <0.05)

## Discussion

As a coronary hemodynamic index, QFR can accurately reflect the changes in coronary blood flow. FAVOR series studies have fully confirmed the accuracy and feasibility of QFR in evaluating coronary blood flow and coronary function [10]. IMR and FFR are gold standards for evaluating coronary microcirculation dysfunction and coronary dysfunction, respectively. However, the current quantitative measurement method of IMR and FFR is complex, and cQFR and cQFR- fQFR are more economical and convenient than FFR and IMR in indicating coronary dysfunction. Lau [11] measured the changes in IMR and CFR after RIPC, confirming the effect of RIPC on coronary microcirculation and hemodynamics. This is consistent with the findings of the present study. Unlike the present study, this study used cQFR with cQFR-fQFR to measure coronary hemodynamics and microcirculatory function, which are more simply and convinient. The cQFR-fQFR in this study showed the same statistical results as the IMR, this may indicate that cQFR-fQFR has similar validity to the IMR in assessing microcirculatory function.To our knowledge, this is the first study to confirm that RIPC can improve coronary function and increase coronary flow by measuring QFR and IMR.

### RIPC increases coronary blood flow and improves coronary microcirculation

There has been conflicting evidence regarding the effect of RIPC on coronary microcirculation. In the LIPSIA-Conditioning trial, four cycles of 30 s reperfusion or post-conditioning failed to improve the myocardial rescue and microvascular occlusion, post-conditioning combined with distal ischemic preconditioning for three cycles of 5-minute upper arm ischemia or 5-minute reperfusion improved myocardial rescue but did not significantly reduce microvascular occlusion [12]. Lau’s study also found that 3 cycles of 5-minute ischemia with 5-minute reperfusion improved coronary blood flow and microcirculation [11].Traverse et al. reported that ischemic postconditioning did not reduce myocardial infarction area; however, reduced microvascular obstruction accelerated the recovery of left ventricular function in patients with STEMI [13]. Therefore different findings may be due to different RIPC protocols.In our study, IMR and c-fQFR were used to verify that RIPC can improve coronary microcirculation in CSX patients for the short term, and the change of cQFR suggested that short-term application of RIPC can increase coronary blood flow.

### Dose effect of RIPC cardioprotection

Although RIPC has been used in clinical settings for many years, no consistent conclusions on its dose-effect exist. Previous studies have reported that the myocardial protective substances released into the plasma after RIPC in one upper limb or one lower limb of healthy volunteers demonstrated no significant difference in their myocardial IRI improvement [14]. Other studies have reported that increasing RIPC tissue mass would enhance its cardioprotective effect [15, 16],and this is consistent with our results.In this study, the improvement of cQFR in the RIPC LL subgroup is more apparent than that in the UL subgroup, suggesting that increasing the tissue mass of RIPC may improve coronary function and increase coronary blood flow more effectively. Although the improvement of IMR and cQFR- fQFR in the LL group was better than that in the UL group after RIPC, no significant difference was observed. This may be because of the small sample size of CSX patients and its inability to fully reflect the statistical relationship, or because the neurohumoral factors that can pass through the coronary microvessels were limited, resulting in a “none or all” effect in the improvement of microcirculation function. In addition, different races and species may require different doses of the stimulus, which may account for the contradictory conclusions obtained from different studies [17, 18]. It is worth noting that increasing the intensity of RIPC may not increase the effect of myocardial protection or even attenuate it, which is known as “hyperconditioning” [19, 20].

### Confounding factors in the cardioprotective effect of RIPC

The results of CONDI-2/ERIPC-PPCI demonstrated that RIPC had no benefit on the long-term clinical outcomes of STEMI patients [21]; however, the RIC-STEMI study reported that RIPC can reduce the combined hard clinical endpoint of cardiac mortality and hospitalization for heart failure in STEMI patients [22]. This is because different races and different schemes of inflation, reperfusion, and circulation cycle may act as confounding factors for the effect of RIPC. In addition, in previous studies, most of the included participants were from economically developed areas who could receive treatment quickly when acute coronary syndrome (ACS) occurs and have improved rehabilitation and medical security after the procedure. However, in economically underdeveloped areas, ACS patients have to tolerate long-term myocardial ischemia before admission. A few studies reported that the cardioprotective effect of RIPC in STEMI patients might increase with the prolongation of ischemic time [23]. Therefore, RIPC may play a vital role in low-income and underdeveloped areas.

Comorbidity and medication are important confounding factors of RIPC because they may affect the response of the myocardium to IRI. Hyperlipidemia, diabetes, and hypertension can increase the myocardial IRI and reduce the cardioprotective effect of RIPC [24, 25]. Some cardiovascular risk factors, including old age and smoking, also weaken the role of RIPC [24]. Regarding medication, long-term use of statins, insulin, metformin, and glucagon-like peptide-1 (GLP-1) analogs have a more significant cardioprotection RIPC [24], whereas the long-term use of glibenclamide [26] and nitrate [27] will reduce or even eliminate its myocardial protective effect. A variety of confounding factors may also be one of the reasons for the different results from different studies. In this study, no significant difference was observed in the abovementioned aspects between the RIPC and control groups.

### Limitations

First, this study was a single-center, small-size study with a population agglomeration, and potential confounding factors and selection bias could not be completely avoided. Third, the treatment effect of RIPC using only one cycle was obtained in the trial, and the long-term use of RIPC could not know the myocardial blood flow status.

## Conclusion

In summary, this study determined that RIPC can increase coronary blood flow and improve coronary microcirculation in the short term, as demonstrated by the cQFR, cQFR-fQFR, and IMR measurements. The short-term application of RIPC increased coronary blood flow in patients with CAD and also improved microcirculatory function, providing a treatment strategy for patients with microcirculatory dysfunction.

## Data Availability

All data generated or analysed during this study are included in this published article.
